# Detecting aspartate isomerization and backbone cleavage after aspartate in intact proteins by NMR spectroscopy

**DOI:** 10.1007/s10858-020-00356-4

**Published:** 2021-01-21

**Authors:** Arthur Hinterholzer, Vesna Stanojlovic, Christof Regl, Christian G. Huber, Chiara Cabrele, Mario Schubert

**Affiliations:** 1 Christian Doppler Laboratory for Innovative Tools for Biosimilar Characterization, University of Salzburg, Hellbrunnerstrasse 34, 5020 Salzburg, Austria; 2 Department of Biosciences, University of Salzburg, Billrothstrasse 11, 5020 Salzburg, Austria; 3 Department of Biosciences, Division of Chemistry and Bioanalytics, University of Salzburg, Hellbrunnerstrasse 34, 5020 Salzburg, Austria

**Keywords:** Post-translational modification, NMR spectroscopy, Monoclonal antibody, N-terminal proline, Peptide bond cleavage

## Abstract

The monitoring of non-enzymatic post-translational modifications (PTMs) in therapeutic proteins is important to ensure drug safety and efficacy. Together with methionine and asparagine, aspartic acid (Asp) is very sensitive to spontaneous alterations. In particular, Asp residues can undergo isomerization and peptide-bond hydrolysis, especially when embedded in sequence motifs that are prone to succinimide formation or when followed by proline (Pro). As Asp and isoAsp have the same mass, and the Asp-Pro peptide-bond cleavage may lead to an unspecific mass difference of + 18 Da under native conditions or in the case of disulfide-bridged cleavage products, it is challenging to directly detect and characterize such modifications by mass spectrometry (MS). Here we propose a 2D NMR-based approach for the unambiguous identification of isoAsp and the products of Asp-Pro peptide-bond cleavage, namely N-terminal Pro and C-terminal Asp, and demonstrate its applicability to proteins including a therapeutic monoclonal antibody (mAb). To choose the ideal pH conditions under which the NMR signals of isoAsp and C-terminal Asp are distinct from other random coil signals, we determined the pK_a_ values of isoAsp and C-terminal Asp in short peptides. The characteristic ^1^H-^13^C chemical shift correlations of isoAsp, N-terminal Pro and C-terminal Asp under standardized conditions were used to identify these PTMs in lysozyme and in the therapeutic mAb rituximab (MabThera) upon prolonged storage under acidic conditions (pH 4–5) and 40 °C. The results show that the application of our 2D NMR-based protocol is straightforward and allows detecting chemical changes of proteins that may be otherwise unnoticed with other analytical methods.

**Supplementary Information:**

The online version of this article (10.1007/s10858-020-00356-4) contains supplementary material, which is available to authorized users.

## Introduction

There is a considerable number of modifications that are observed in therapeutic proteins, including tightly controlled enzymatic modifications (e.g., phosphorylation and glycosylation) and spontaneously occurring modifications (e.g., pyroglutamate and succinimide formation, oxidation) (Grassi et al. 2017). Two spontaneous modifications of proteins including therapeutic mAbs are the isomerization of Asp to isoAsp and the non-enzymatic cleavage of the Asp-Xaa peptide bond (Diepold et al. 2012; Harris et al. 2001). Whereas spontaneous cleavage is critically influencing the stability of therapeutic proteins (Vlasak and Ionescu 2011), isomerization of Asp can lead to loss of potency and efficacy (Cacia et al. 1996; Harris et al. 2001; Rehder et al. 2008; Wakankar et al. 2007a; Yan et al. 2016) and even trigger an undesired immunological response (Doyle et al. 2006; Yang et al. 2006). The most sensitive peptide bond prone to spontaneous cleavage was reported to be Asp-Pro (Landon 1977; Piszkiew et al. 1970) with half-lives of months (Li et al. 2009). However, cleavage of other Asp-Xaa bonds was also observed (Vlasak and Ionescu 2011), for example of Asp-Cys (Pane et al. 2016). The cleavage of Asp-Xaa bonds is facilitated, in general, under acidic conditions (Landon 1977; Li et al. 2009; Marcus 1985; Piszkiew et al. 1970; Vlasak and Ionescu 2011). Accordingly, the mechanism of Asp-Xaa peptide-bond cleavage starts with an acid-catalyzed intraresidue nucleophilic attack of the side-chain carboxylate at the backbone carbonyl group, forming a cyclic anhydride intermediate together with the cleavage of the peptide bond, as derived from NMR spectroscopy and isotopic labeling experiments, as well as the analysis of cross-linking products of the reactive anhydride (Joshi et al. 2005; Oliyai and Borchardt 1993; Wang et al. 2019). As a result, the amino acid succeeding Asp is released as new N-terminus, whereas the anhydride intermediate is converted into a C-terminal Asp residue upon water attack at either of the two carbonyl groups. Asp-Xaa peptide-bond cleavage can be also observed as a side reaction of protein splicing, when an Asp is flanking the spliced-out intein (Minteer et al. 2017).

Isomerization of Asp to isoAsp is an important degradation mechanism in proteins and in particular mAbs (Cacia et al. 1996; Lu et al. 2019; Rehder et al. 2008; Wakankar et al. 2007a), which involves succinimide (Snn) formation as an intermediate as proven by ^18^O labeling (Wang et al. 2007). Unlike the Asp-Xaa cleavage, which requires an intraresidue acid-catalyzed cyclization, the isomerization of Asp to isoAsp involves an interresidue cyclization via the nucleophilic attack by the α-nitrogen of the residue following Asp at the side-chain carboxylate of Asp, resulting in Snn formation upon water elimination (Johnson et al. 1989). Snn is usually stable under mildly acidic conditions (Grassi et al. 2017; Tomizawa et al. 1994), but at neutral to basic pH it is readily hydrolyzed to a mixture of Asp and isoAsp in a ratio of about 1:3 (Geiger and Clarke 1987; Johnson et al. 1989). Although Snn formation is mainly associated with deamidation of Asn residues (Capasso et al. 1995; Geiger and Clarke 1987; Robinson et al. 1970), isomerization of Asp to isoAsp via Snn is important as well. Both Asn deamidation and Asp isomerization occur preferentially at Asx-Gly, Asx-Ser, Asx-Ala and Asx-His (Asx = Asp or Asn) under neutral or mildly acidic conditions (Robinson and Robinson 2001; Yi et al. 2013). However, isomerization at Asp-Asp and Asp-Tyr have been observed in mAbs as well (Kern et al. 2014; Rehder et al. 2008; Yi et al. 2013). Typically, this isomerization is very slow with rates that correspond to half-lives of weeks to years as determined at 37 °C (Li et al. 2009) or few days at 50 °C (Wakankar et al. 2007a, b), both studied under a variety of pH conditions.

Furthermore, the primary structure, local solvent accessibility, and flexibility within a folded protein have a critical influence on deamidation and Asp-isomerization rates (Harris et al. 2001; Wakankar et al. 2007a) and can also influence the ratio of the different products. In unstructured peptides, there is typically an equilibrium between Snn, Asp, and isoAsp (Geiger and Clarke 1987). There is a small number of NMR studies of proteins containing isoAsp of folded, recombinant and mostly ^13^C/^15^N labeled proteins, with complete backbone assignments (Chazin et al. 1989; Mallagaray et al. 2019; Revington and Zuiderweg 2004; Rogov et al. 2003; Tugarinov et al. 2002; Wong et al. 2020), whereas not a single NMR study of a folded protein containing Snn is available. However, few examples of protein crystal structures containing Snn can be found in the Protein Data Bank, and the random coil chemical shifts of Snn within peptides have been reported (Grassi et al. 2017). In contrast, the occurrence of isoAsp as a result of isomerization in proteins is probably underestimated as isoAsp is not easily observable by the primary analytical tool used for protein characterization (LC–MS).

Traditionally isoAsp was detected (indirectly) with Edman sequencing, which stops at isoAsp sites (Zhang et al. 2002), but this is not an explicit proof for isoAsp. Another approach involves the enzyme protein-l-isoaspartyl methyltransferase, which specifically methylates isoAsp at its α-carboxyl group leading to a mass difference of 14 Da. However, the methylated isoAsp tends to spontaneously cyclize back to Snn, which in turn is in equilibrium with Asp and isoAsp due to hydrolysis. Furthermore, digestion with Asp-N is used to indirectly localize isoAsp, as Asp-N cleaves N-terminal of Asp but not isoAsp (Zhang et al. 2002).

IsoAsp- and Snn-containing variants of mAbs and their digested peptides could be successfully separated using liquid chromatography like hydrophobic interaction chromatography (HIC) (Cacia et al. 1996; Dick et al. 2009; Eakin et al. 2014), or reversed-phase chromatography (RPC), but for the identification of the signals either synthetic reference peptides are required (Yi et al. 2013) or sophisticated HPLC–MS techniques (Sze et al. 2020) or capillary zone electrophoresis (Bergstrom et al. 2015) have to be applied. In peptides, isoAsp can be distinguished from Asp by ESI-MS^2^, which shows differences in the intensity ratios of the complementary b and y ions (Lehmann et al. 2000). Also, fragmentation reactions involving electron capture dissociation (ECD) and electron transfer dissociation (ETD) are used to distinguish isoAsp from Asp with MS (Cournoyer et al. 2005; DeGraan-Weber et al. 2016; Ni et al. 2010; O'Connor et al. 2006). In any case, a new and orthogonal method to independently detect isoAsp is desireable for cross-validation.

Cleavage of peptide bonds was traditionally detected by SDS-PAGE (Lamed et al. 2001; Lidell and Hansson 2006), and the exact localization of the cleavage was conventionally achieved by Edman sequencing (Lamed et al. 2001; Lidell and Hansson 2006), nowadays by more sophisticated MS techniques (Liu et al. 2008; Osicka et al. 2004). In MS of intact proteins sometimes a mass difference of + 18 Da is observed, which, however, cannot be unambiguously assigned to the cleavage of a peptide bond. Indeed, only under denaturing and reducing conditions the fragments can be separated, and analysis by MS will reveal the cleavage site, which can be further supported by peptide mapping and sequencing by MS^2^ (Liu et al. 2008). Asp-Pro cleavage has also been reported for mAbs, in particular at Asp270-Pro271 or Asp272-Pro273 in the heavy chain of the Fc part of human immunoglobulin gamma 1 (IgG1) (Davagnino et al. 1995; Rehder et al. 2006; Vlasak and Ionescu 2011) and 2 (IgG2) (Van Buren et al. 2009), respectively.

Nevertheless, quantification of peptide bond cleavage is challenging, especially due to the risk that additional cleavage can occur during the MS measurement (Kim et al. 2013; Maux et al. 2002; Takayama 2016). Therefore complementary methods are of great interest.

Here we report a straightforward NMR approach for the unambiguous detection of both isoAsp formation and backbone cleavage after Asp. With a detailed NMR investigation, we identified characteristic chemical shift correlations to unambiguously detect and potentially quantify these modifications in intact proteins, as illustrated by lysozyme and the biotherapeutic mAb rituximab.

## Results

### Random coil chemical shifts of C-terminal Asp and N-terminal Pro

**Fig. 1:**
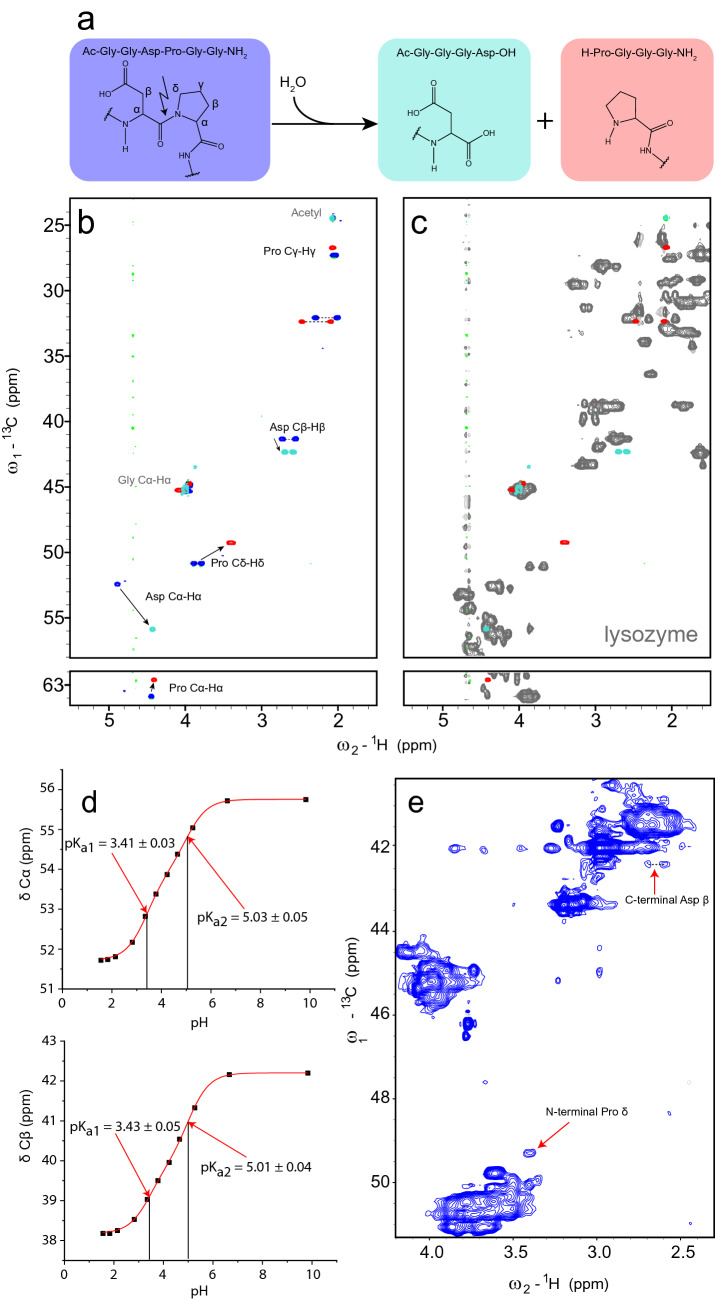
Random coil chemical shift correlations of lysozyme and model peptides for Asp-Pro peptide bond cleavage. **a** Scheme of the cleavage reaction. **b** Overlaid ^1^H-^13^C HSQC spectra of the three peptides Ac-Gly-Gly-Asp-Pro-Gly-Gly-NH_2_, H-Pro-Gly-Gly-Gly-NH_2_ and Ac-Gly-Gly-Gly-Asp-OH recorded under denaturing conditions (7 M urea-d_4_ + D_2_O) at pH 7.4. **c** Comparison of the ^1^H-^13^C HSQC spectra of the two reference peptides (H-Pro-Gly-Gly-Gly-NH_2_, Ac-Gly-Gly-Gly-Asp-OH) with denatured lysozyme (grey) to identify unique random coil chemical shifts suitable for the detection of Asp_C-term_ and Pro_N-term_. **d** Dependence of the chemical shifts of Asp_C-term_ on the pH. Data were fitted with a modified Henderson Hasselbalch equation (Eqs. 3 or 4) for extracting two pK_a_ values. The resulting pK_a_ values are given. **e** ^1^H-^13^C HSQC fingerprint spectrum of rituximab (incubated for 138 h at pH 4 and 40 °C) shows specific cross peaks for Cβ-Hβ of C-terminal Asp and Cδ-Hδ of Pro_N-term_ (measurement conditions: 7 M urea-d_4_ in D_2_O, pH 7.4, 600 MHz, cryo probe, 96 scans, 512 × 256 complex points, 86 h measurement time)

To develop an approach for the detection of Asp-Pro cleavage (Fig. 1a), we hypothesized that the new terminal ends, which are formed after peptide bond cleavage, would display characteristic random coil chemical shifts. A complete assignment of a C-terminal Asp (Asp_C-term_) and N-terminal Pro (Pro_N-term_) within small synthetic peptides was obtained under denaturing conditions (7 M urea-d_4_ in D_2_O) at the pH values 2.3 and 7.4 (Tables 1 and S1) using ^1^H-^13^C HSQC, ^1^H-^1^H TOCSY, ^1^H-^13^C HMBC, and ^1^H-^13^C HMQC-COSY spectra. The two pH conditions were chosen based on previous work on random coil chemical shifts of PTMs, the acidic condition (pH 2.3) for aiding denaturation, the neutral condition (pH 7.4) for fragile moieties. When compared with the random coil chemical shifts of the 20 natural amino acids, we noticed a characteristic cross peak of Cδ–Hδ at 49.3 ppm and ~ 3.4 ppm (Fig. 1b) for the Pro_N-term_, which is separated from other random coil chemical shifts (Fig. 1c). As expected, the random coil chemical shifts of Pro_N-term_ did not change much between the two tested pH values (Table 1, Fig. S1). Therefore, also pH values between 2.3 and 7.4 will be suitable for the detection of Pro_N-term_. In the case of Asp_C-term_, the Cβ and Hβ random coil chemical shifts change with pH (Fig. S2), which is mainly due to the ionization state of the side-chain carboxyl group (neutral at pH 2.3 and negatively charged at pH 7.4) and, to a smaller extent, to the ionization state of the C-terminal carboxyl group. Whereas the Cβ–Hβ correlations of Asp_C-term_ at pH 2.3 overlap with common random coil chemical shifts (Fig. S2a), the Cβ–Hβ correlations at pH 7.4 are surprisingly distinct from other random coil chemical shifts of Asp within a peptide or protein chain (Fig. S2b). To further study the pH dependence of the chemical shifts, we also measured spectra at different pH values ranging from 1.6 to 9.8 (Fig. 1d, Fig. S3 and Table S2). The data could be fitted using modified Henderson-Hasselbalch equations that account for two titration events resulting in the pK_a_ values of 3.2 and 5.0. Due to the larger chemical shift changes for Hα and Cα around pH 3.2, and for Cβ and Hβ around pH 5.0, the first and second pK_a_ values were assigned to the α- and β-carboxyl group, respectively.

**Table 1:** Random coil chemical shifts of the peptides containing an Asp_C-term_ and a Pro_N-term_ together with a peptide containing the Asp-Pro motif in 7 M urea-d_4_, D_2_O at two pH values (2.3, 7.4)

Peptide	Ac-Gly-Gly-Gly-Asp-OH		H-Pro-Gly-Gly-Gly-NH_2_		Ac-Gly-Gly-Asp-Pro-Gly-Gly-NH_2_			
pH	2.3	7.4	2.3	7.4	2.3	7.4	2.3	7.4
Residue	Asp_C-term_		Pro_N-term_		Asp		Pro	
C	177.3^b^	181.0^b^	173.0	173.4	173.2	175.1	177.5	177.7
Cα	52.0	55.9	62.6	62.6	51.2	52.4	63.9	63.9
Cβ	38.4	42.3	32.4	32.4	38.0	41.3	32.0	32.1
Cɣ	177.5^b^	181.5^b^	26.7	26.7	177.0	180.1	27.4	27.3
Cδ	–	–	49.3	49.3	–	–	50.7	50.9
Hα	4.79	4.43	4.44	4.41	5.00	4.89	4.43	4.45
Hβ2^a^	2.97	2.69	2.49	2.47	2.94	2.73	2.29	2.30
Hβ3^a^	2.97	2.59	2.11	2.09	2.76	2.56	2.00	2.01
Hγ2	–	–	2.08	2.07	–	–	2.07	2.04
Hγ3	–	–	2.08	2.07	–	–	2.02	2.04
Hδ2	–	–	3.44	3.41	–	–	3.77	3.89
Hδ3	–	–	3.41	3.39	–	–	3.74	3.79

^a^Not stereochemically assigned, the lower value was tentatively assigned to Hβ3

^b^These overlapping resonances might be swapped

### Induction and detection of the cleavage between Asp and Pro at the peptide and protein level

Asp-Pro cleavage was initially studied with the model peptide Ac-Gly-Gly-Asp-Pro-Gly-Gly-NH_2_ that was incubated for 25 h in D_2_O at 60 °C and at pH 2.6. A ^1^H-^13^C spectrum recorded before and after incubation is shown in Fig. S4. After 25 h approx. 85% of the peptide was already cleaved as judged from the integrals of the cross-peaks.

To induce a detectable amount of Asp-Pro cleavage in a large protein, we chose the therapeutic mAb rituximab, which was incubated in 250 mM ammonium acetate buffer (pH 4) at 40 °C for 138 h. The only Asp-Pro motif in the sequence of rituximab is in the Fc region (Fig. S5). The ^1^H-^13^C HSQC spectrum (Fig. 1e) measured under denaturing conditions at pH 7.4 shows characteristic signals of Pro_N-term_ as well as Asp_C-term_ (Table S6), which unambiguously shows the presence of Asp-Pro cleavage in the treated sample. Investigation with HPLC–MS detected cleavage products confirming the presence of the cleavage between Asp274 and Pro275 as the predominant one (Fig. S6, Table S3).

Also, recombinant Fc/2 protein, which we studied earlier in the context of succinimide formation (Grassi et al. 2017), showed the characteristic signal Cδ-Hδ of Pro_N-term_ after treatment at pH 4 (Fig. S7). Since the spectrum of Fc/2 was measured at pH 2.3, we could not detect the characteristic Cβ-Hβ correlations of Asp_C-term_ due to signal overlap at this pH value, but the Cα-Hα correlation indicates the presence of Asp_C-term_. The occurrence of backbone cleavage was confirmed by MS (Fig. S7 d, Table S4).

### Unique random-coil chemical shifts of isoAsp

**Fig. 2:**
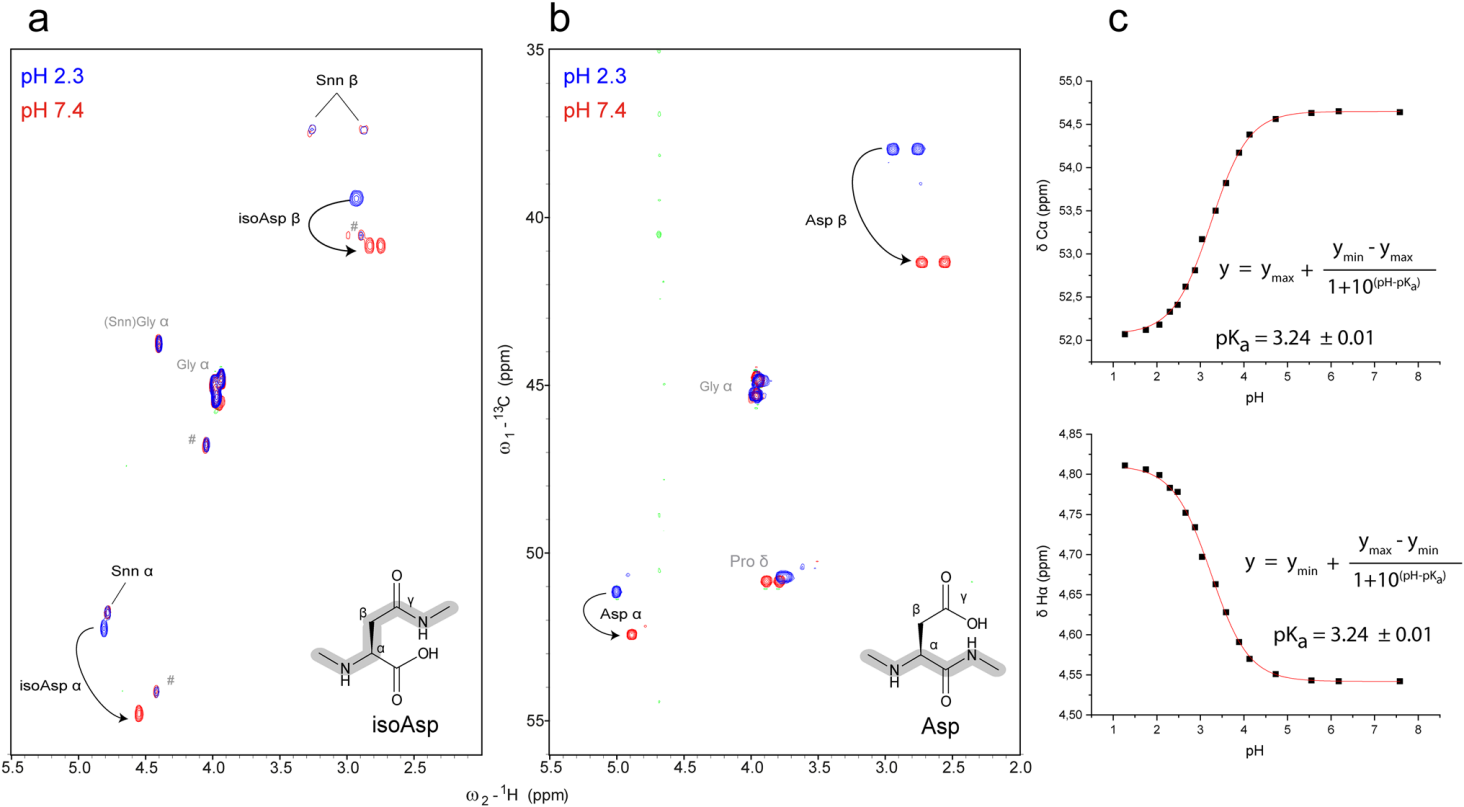
Overlay of the reference peptides containing Asp and isoAsp in protonated and deprotonated form. **a** Overlay of ^1^H-^13^C HSQC spectra of the peptide Ac-Gly-Gly-isoAsp-Gly-Gly-NH_2_ at the two pH values 2.3 (blue) and 7.4 (red). Furthermore Snn can be detected due to the equilibrium between isoAsp and Snn. ^#^ impurity of cyclo(Gly-Asp) diketopiperazine peptide, whose signals are pH independent. **b** Overlay of ^1^H-^13^C HSQC spectra of the peptide Ac-Gly-Gly-Asp-Pro-Gly-Gly-NH_2_ at the two pH values 2.3 (blue) and 7.4 (red). As Asp has typically a pK_a_ value of approx. 3.9 (Platzer et al. 2014), the random coil chemical shifts of protonated and deprotonated Asp change significantly. **c** Dependence of the chemical shifts of isoAsp on the pH. Data were fitted with a modified Henderson Hasselbalch equation (Eqs. 3 or 4) for extracting the pK_a_ value. The resulting pK_a_ values are given

In previous work, we observed that the chemical shift correlations of isoAsp at pH 2.3 coincided with typical random coil correlations of the 20 common amino acids (Grassi et al. 2017). Therefore, we decided to change the ionization state of isoAsp aiming to obtain characteristic signals. For this reason, we recorded ^1^H-^13^C HSQC spectra of the isoAsp-containing peptide Ac-Gly-Gly-isoAsp-Gly-Gly-NH_2_ at pH 2.3 as well as pH 7.4 (Fig. 2). As anticipated above, the comparison between the two conditions showed large changes in the chemical shifts of isoAsp (Table 2), hinting that there might be conditions at which the chemical shift correlations are unique. To judge the influence of small pH variations on the chemical shifts and the suitability of certain chemical shift correlations, we measured ^1^H-^13^C HSQC spectra at different pH values ranging from 1.27 to 7.6 (Table S5). All chemical shifts of isoAsp changed during the titration. The pH dependence of the chemical shifts (Figs. 2c and S8) was fitted using the Henderson-Hasselbalch equation (Eq. 3,4) to determine the pK_a_ value of isoAsp (Table 3, Figs. 2c and S8). We determined a value of 3.2. Although we measured the pK_a_ in D_2_O with direct reading of the H_2_O-calibrated pH-meter (pK_a_^H*^), it was noticed previously that pK_a_^H*^ measurements in D_2_O are just about 0.06 pH units higher than pK_a_ values measured in H_2_O (pK_a_^H^), at least under acidic conditions (Bundi and Wüthrich 1979). In a more recent study deviations of < 0.2 pH units were observed between pK_a_^H*^ and pK_a_^H^ for carboxyl groups in short peptides (Krezel and Bal 2004).

**Table 2:** Random coil chemical shifts (ppm) of isoAsp and Snn in small model peptides in 7 M urea-d_4_, D_2_O at two pH values (2.3, 7.4)

Peptide	Ac-Gly-Gly-IsoAsp-Gly-Gly-Gly-NH_2_				Previously reported values^c^	
Amino acid	isoAsp		Snn^b^		isoAsp	Snn
pH	2.3	7.4	2.3	7.4	2.3	2.3
C	177.0	179.5	180.0	n.d	177.0	179.5
Cα	52.3	54.8	51.8	51.8	52.3	52.0
Cβ	39.4	40.8	37.4	37.4	39.5	37.5
Cɣ	175.5	176.5	n.d	n.d	175.3	179.9
Hα	4.81	4.55	4.78	4.78	4.79	4.78
Hβ2^a^	2.93	2.83	3.26	3.27	2.92	3.26
Hβ3^a^	2.93	2.75	2.87	2.86	2.92	2.87

^a^β2 and β3 chemical shifts are not sterochemically assigned, the lower value was tentatively assigned to Hβ3

^b^Small amounts of Snn were detected (~ 35%) due to its equilibrium with isoAsp; n.d. not determined

^c^Random coil chemical shifts reported earlier (Grassi et al. 2017) measured at pH 2.3 with the peptides Ac-Glu-Trp-Ser-isoAsp-Gly-Gln-Pro-Glu-Asn-NH_2_ and Ac-Glu-Trp-Ser-Snn-Gly-Gln-Pro-Glu-Asn-NH_2_

The titration data revealed that neutral pH seems to be ideally suitable to unambiguously detect Cβ-Hβ correlations of isoAsp, because the chemical shifts do not depend on small pH changes and the signals do not overlap with the random coil chemical shifts of natural amino acids.

**Fig. 3:**
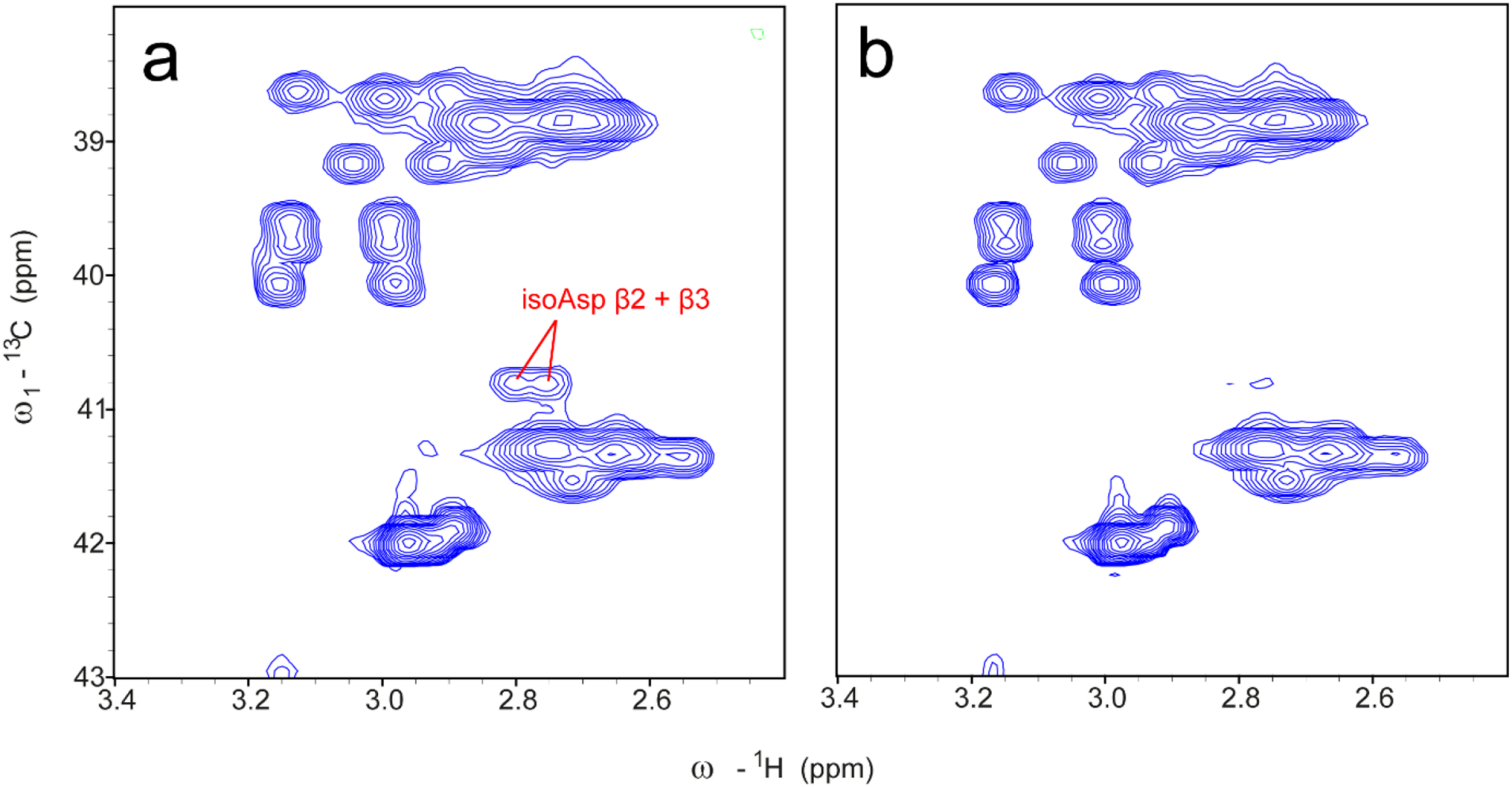
Detection of isoAsp in incubated lysozyme showing the unique Cβ-Hβ chemical shift correlations of isoAsp in a ^1^H-^13^C HSQC spectrum at pH 7.4 under denaturing conditions. **a** The ^1^H-^13^C HSQC spectrum of lysozyme (concentration: 36 mg/mL) incubated for 10 days at pH 4 and 40 °C recorded with 120 scans (3 days measurement time) and 512 × 512 complex points. The isoAsp Cβ–Hβ correlations are well isolated and suited for detection and quantification. As a negative control, panel **b** shows a ^1^H ^13^C HSQC spectrum of non-treated lysozyme (concentration: 36 mg/mL, 120 scans, 512 × 512 complex points, 3 days measurement time)

The characteristic signals of isoAsp could be detected in a ^1^H-^13^C HSQC spectrum of denatured lysozyme that was incubated at pH 4 for 138 h (Fig. 3a). These signals were absent in the spectrum of untreated lysozyme (Fig. 3b). This example illustrates that a ^1^H-^13^C HSQC fingerprint spectrum can identify (unambiguously) isoAsp in denatured proteins.

## Discussion

Our long-term goal is to use a ^1^H-^13^C HSQC spectrum of a biotherapeutic protein to identify and quantify spontaneous or enzymatic modifications with a single experiment using characteristic chemical shifts correlations measured under denaturing conditions. Such an initial screening of prospective biotherapeutics treated under induced degradation conditions will be enormously valuable to identify any potential modification that can occur during processing, formulation, and storage. This is valuable information, because many analytical techniques, especially MS, typically require that the modifications to be monitored are known beforehand. In contrast, NMR spectroscopy can also identify less-established (Schweida et al. 2019) or completely unknown modifications.

The NMR assignments presented here for C-terminal Asp, N-terminal Pro, and internal isoAsp residues under denaturing conditions at pH 2.3 and 7.4 are suited to detect the cleavage of Asp-Xaa peptide bonds in denatured proteins (excepted proteins with Xaa and Asp as amino- and carboxyl termini, respectively), and to identify isoAsp resulting from either Asn deamidation or Asp isomerization. For C-terminal Asp, the key correlations of Cβ-Hβ are unique at pH 7.4 and in this case do not overlap with the random coil chemical shifts of the 20 natural amino acids. Therefore, the Cβ-Hβ at pH 7.4 is well suited to unambiguously identify and quantify peptide bond cleavage between Asp and any following amino acid (Asp-Xaa). An Asp-Pro cleavage can be further identified by a unique ^1^H-^13^C correlation of Cδ-Hδ of the Pro_N-term_, which is not influenced by the pH. For isoAsp, the Cβ-Hβ random coil chemical shift correlations are well-separated from those of Asp at pH 7.4, and they can be used to identify and potentially quantify isoAsp in intact proteins under denaturing conditions.

The pK_a_ values of isoAsp and Asp_C-term_, which we determined for choosing appropriate pH conditions, will be also valuable for other analytical techniques like capillary electrophoresis, for a better understanding of the role of electrostatic effects in enzyme mechanisms, molecular modeling, and more advanced theoretical approaches. The pK_a_ value of the isoAsp side chain was determined to be 3.2 (Table 3, Figs. 2c, S8), which is 0.7 pH units lower than the pK_a_ value reported for an Asp side chain (approx. 3.9) (Platzer et al. 2014). The two pK_a_ values of Asp_C-term_ (Table 3) were 3.4 for the backbone carboxyl group and 5.0 for the side chain. Interestingly, the acidity of the α-carboxyl group is the same for internal isoAsp and C-terminal Asp, whereas the acidity of the β-carboxyl group is significantly lower in a C-terminal Asp than in an internal Asp residue (5.0 versus approx. 3.9). This is in agreement with an earlier report, where a pK_a_ value of 4.5 was measured for the side chain of an internal Asp flanked by two Glu residues in an intrinsically disordered protein, which was explained with the presence of other negatively charged groups in the proximity (Neira et al. 2016).

**Table 3:** pK_a_ values of isoAsp and C-terminal Asp in peptides reported in Table 1 in comparison with previously reported values (Platzer et al. 2014).

	Experimental pK_a_ (this work)^a^	Previously reported pK_a_ values^b^
isoAsp (internal)	3.2	
Asp (internal)		3.86
Asp_C-term_ (side chain)	5.0	
Asp_C-term_ (carboxyl terminus)	3.4	
Ala_C-term_		3.55

^a^pK_a_^H*^ values measured in D_2_O with direct reading of the H_2_O-calibrated pH-meter, at 0 mM NaCl

^b^pK_a_^H^ values measured in H_2_O at 50 mM NaCl

## Conclusion

We demonstrate here the application of 2D NMR spectroscopy to unambiguously identify protein degradation products derived from Asp-Xaa peptide-bond cleavage, Asp isomerization, or Asn deamidation in any protein that can be denatured. There is no limit concerning the protein size as long as the denatured protein remains in solution, and the straightforward approach can be applied to any modern NMR instrument with medium to high field and two or more channels. Considering the importance of ensuring the safety and efficacy of therapeutic proteins, unambiguous identification of all potentially occurring modifications is crucial. Although not suitable for high-throughput routine applications, our NMR approach is ideal for cross-validation purposes, in combination with MS-based characterization of biotherapeutics.

## Experimental section

### Chemicals for peptide synthesis

Chemical reagents and solvents for the peptide synthesis were of peptide-synthesis grade; solvents for HPLC were of HPLC grade. Fmoc-protected amino acids, Rink-amide MBHA resin (100–200 mesh, loading 0.57 mmol/g), *N*,*N*-diisopropylethylamine (DIPEA), piperidine, *N*,*N*-dimethylformamide (DMF), *N*-methyl-2-pyrrolidone (NMP), dichloromethane (DCM), diethyl ether, and trifluoroacetic acid (TFA) were purchased from Iris Biotech (Germany). H-Asp(OtBu)-2-chlorotrityl resin (loading 0.60 mmol/g) was purchased from Merck Schuchardt OHG (Germany). Thioanisole (TIA), acetic anhydride, α-cyano-4-hydroxycinnamic acid, triisopropylsilane (TIS), 1,2-ethanedithiol (EDT), and acetonitrile (ACN) were purchased from Sigma Aldrich (Germany). 2-(1H-benzotriazole-1-yl)-1,1,3,3-tetramethyluronium hexafluorophosphate (HBTU) and *N*-hydroxybenzotriazole (HOBt) were purchased from Biosolve (The Netherlands).

### Solid-phase peptide synthesis

Peptides (Table S1) were synthesized by Fmoc-chemistry using solid-phase peptide synthesis (SPPS) on an automatic peptide synthesizer (Syro I, Biotage). The resin-bound peptides were cleaved and deprotected with TFA containing 10% scavenger mixture H_2_O/TIA/EDT/TIS (1:3:3:3) at room temperature for 1.5 h. The peptides were then precipitated from cold diethyl ether, recovered by centrifugation at 4 °C, washed three times with cold ether, dried under nitrogen, dissolved in 0.1% aqueous TFA, and lyophilized.

### Peptide characterization

Analytical RP‐HPLC was performed using a Thermo Scientific™ Dionex™ UltiMate™ 3000 UHPLC system (Thermo Fisher Scientific, Germering, Germany) and a Syncronis C‐18 column (100 Å, 5 μm, 250 × 4.6 mm, Thermo Fisher Scientific) at a flow rate of 1.5 mL/min. The UV detection was set at 220 nm. The elution system was (A) 0.06% (v/v) TFA in water, and (B) 0.05% (v/v) TFA in ACN. The peptides were dissolved in ACN/H_2_O (10:90, v/v) containing 0.1% TFA. Analytical chromatograms were obtained with the following gradient: 1% B for 8 min, then to 50% B in 35 min. Mass spectra were recorded on an Autoflex Speed MALDI‐TOF mass spectrometer (Bruker Daltonics, Bremen, Germany) by using α‐cyano‐4‐hydroxycinnamic acid as matrix.

### Sample treatment and preparation

#### Rituximab and reference peptides

For rituximab, the buffer of 2 mL formulation solution of Mabthera (Mabthera, Roche; exp. year: 2013, 2 mL, 10 mg/mL) was exchanged to 0.25 M ammonium acetate buffer (pH 4) with Amicon Ultra-15 Centrifugal Filter Units (Cutoff: 30 kDa, Merck) and the sample was incubated for 138 h at 40 °C. Afterward, the buffer was changed to ddH_2_O overnight with a Spectra/Por dialysis membrane and lyophilized. For NMR measurements the sample was dissolved in 500 μL of a 7 M urea-d_4_ (98 atom%D, ARMAR Chemicals) solution in D_2_O (100 at.% D, ARMAR Chemicals) resulting in a concentration of 30 to 40 mg/mL mAb. Urea solutions were freshly prepared to minimize the formation of isocyanic acid which leads to carbamylation of lysine residues. For reducing the disulfide bonds, approx. 1–1.6 mg of tris(2-carboxyethyl)phosphine hydrochloride (TCEP) (Sigma-Aldrich) was added to the sample corresponding approximately to 11 mmol L^−1^ followed by incubation at 60 °C for 15 min. The pH was adjusted to 2.3 or 7.4 by adding DCl or NaOD (ARMAR Chemicals), respectively.

1 to 2 mg of each reference peptide (Table 2) was dissolved at the same conditions as full-length rituximab but without TCEP treatment. Afterward, the pH was adjusted to 2.3 or 7.4 by adding DCl or NaOD (ARMAR Chemicals), respectively.

#### Lysozyme

To induce the formation of isoAsp, 75 mg lysozyme powder from chicken egg white (L4919, Sigma-Aldrich) was dissolved in 50 mL 0.5 M ammonium acetate (pH adjusted to 4) and incubated for 10 days at 40 °C. Afterward, the buffer was changed to ddH_2_O via dialysis with a Spectra/Por dialysis membrane (cutoff 3.5 kDa) and lyophilized. For one NMR sample an amount of 18 mg of treated or non-treated lysozyme was dissolved in 500 μl 7 M urea-d_4_ (98 atom%D, ARMAR Chemicals) solution in D_2_O (100 atom%D, ARMAR Chemicals). For reducing disulfide bonds, 1–1.6 mg TCEP was added and the sample was incubated at 60 °C for 15 min. The pH was adjusted to 2.3 (DCl, ARMAR Chemicals) or 7.4 (NaOD, ARMAR Chemicals), respectively.

#### Mass spectrometry

The lysozyme and recombinant Fc/2 samples were diluted in 0.10% (v/v) aqueous formic acid (FA) to a final concentration of 0.50 mg^.^mL^−1^. Intramolecular disulfide bonds were reduced with 5 mmol L^−1^ tris (2-carboxyethyl) phosphine hydrochloride (TCEP) at 60 °C for 30 min. Five microliters of sample were injected in in-line split-loop mode on the HPLC system described earlier (Grassi et al. 2017), using a Waters XBridge Protein BEH C4 column (150 × 2.1 mm i.d., 3.5 μm particle size, 300 Å pore size) operated at a column temperature of 60 °C. Mobile phase A was H_2_O + 0.10% FA, mobile phase B was composed of acetonitrile (ACN) + 0.10% FA, the applied flow rate was 200 μL min^−1^. The separation was performed with an initial equilibration at 5.0% B for 5 min, followed by a linear gradient of 5.0−50.0% B in 20 min, column regeneration at 99.99% B for 10 min, and re-equilibration at 5.0% B for 15 min. Mass spectrometry was conducted on a Thermo Scientific™ Q Exactive™ Hybrid Quadrupole-Orbitrap™ mass spectrometer equipped with an Ion Max™ source with a heated electrospray ionization (HESI) probe. The instrument settings were as follows: source heater temperature of 200 °C, spray voltage of 3.5 kV, sheath gas flow of 20 arbitrary units, auxiliary gas flow of 5 arbitrary units, capillary temperature of 250 °C, S-lens RF level of 70.0, in-source CID of 20.0 eV, AGC target of 1e6 and maximum injection time of 200 ms. The measurements were carried out in full scan mode with a range of *m/z* 500–2500 at a resolution setting of 140,000 at *m/z* 200.

Rituximab drug product sample was diluted to a concentration of 5.0 mg mL^−1^ in 175 mmol L^−1^ ammonium acetate. Subsequently, disulfides were reduced in 4 mol L^−1^ GdnHCl with 5 mmol L^−1^ TCEP for 15 min at 60 °C. Rituximab was analyzed employing the same HPLC–MS set up with the same mobile phases but different settings: The column used for separation was a Thermo Scientific™ MAbPac™ RP column (150 × 2.1 mm i.d., 4.0 μm particle size,∼1500 Å pore size) operated at 70 °C and a flow rate of 200 μL min^−1^. The gradient applied was the following: 10.0% B for 2 min, 20.0 − 35.0% B in 10.5 min, 80% B for 2.5 min, and 10.0% B for 10 min. The instrument settings of the mass spectrometer were as follows: spray voltage of 3.5 kV, sheath gas flow of 15 arbitrary units, auxiliary gas flow of 5 arbitrary units, in-source CID of 40.0 eV, capillary temperature of 300 °C, S-lens RF level of 80.0, AGC target of 1e6, and maximum injection time of 200 ms. The measurements were carried out in full scan mode with a range of *m/z* 1000−4000 at a resolution setting of 140,000 at *m/z* 200. For deconvolution of raw mass spectra into zero charge-state spectra the Xtract algorithm integrated into the Thermo Scientific™ BioPharma Finder™ software version 4.0 was used.

#### NMR spectroscopy

Spectra were recorded on a 600 MHz Bruker Avance III HD spectrometer equipped with a ^1^H/^13^C/^15^N/^31^P quadruple-resonance room temperature probe at 298 K, except incubated rituximab that was measured on a 600 MHz Bruker Avance III HD spectrometer equipped with a cryogenetic ^1^H/^13^C/^15^N/^31^P quadruple-resonance probe (QCI). For all the NMR measurements, standard 5 mm NMR tubes (ARMAR, Type 5TA) were used with a sample volume of 500 μL. The HSQC fingerprint spectra of the reference peptides were assigned using the following 2D experiments: ^1^H-^13^C HSQC, ^1^H-^13^C HMBC (hmbcgpndqf), ^1^H-^1^H TOCSY, ^1^H-^1^H COSY (cosygpppqf), ^1^H-^13^C HMQC-COSY, ^1^H-^1^H ROESY, ^1^H-^15^N HSQC, and ^1^H-^13^C HCO (Grassi et al. 2017). For measuring and processing the Data, Topspin 3.5/3.6.1 (Bruker) was used. Sparky 3.114 (T. D. Goddard and D. G. Kneller, SPARKY 3, University of California, San Francisco, USA) was used for analyzing the NMR data.

For referencing, 2,2-dimethyl-2-silapentane-5-sulfonic acid (DSS) (ARMAR Chemicals) was added to the samples after measuring the initial spectra. A 1D ^1^H experiment was performed for referencing the proton chemical shift. The carbon and nitrogen dimensions were referenced according to the IUPAC-IUB recommended chemical shifts referencing ratios of 0.251449530 (^13^C) and 0.101329118 (^15^N) (Markley et al. 1998). Chemical shift assignments of all peptides were deposited in the BioMagResBank (Ulrich et al. 2008) under accession numbers 50598, 50599, 50600 and 50601.

### pK_a_ determination of isoAsp and Asp_C-term_

For the determination of the pK_a_ values of isoAsp and Asp_C-term_, 2 mg of the peptides Ac-Gly-Gly-isoAsp-Gly-Gly-NH_2_ or Ac-Gly-Gly-Gly-Asp-OH were dissolved in 500 µl D_2_O. For referencing, 5 µl (100 mM) DSS solution was added. The pH was stepwise adjusted with 0.1 to 1 M NaOD or 0.1 to 1 M DCl (Tables S2, S5). The pH values were measured at room temperature (approx. 25 °C) and the pH meter (EL20 pH-meter, Mettler Toledo) with the pH electrode (MiniTrode, Hamilton) was calibrated using fresh standards at pH 4.00 and 7.00 (AVS TITRINORM, VWR Chemicals). For all of the pH steps, ^1^H 1D and ^1^H-^13^C spectra were measured. The chemical shifts of isoAsp and Asp_C-term_ plotted as a function of pH were fitted using the modified Henderson-Hasselbalch equations (Eqs. 1, 2, 3, and 4) (Farrell et al. 2010; Silverstein 2012) yielding the pK_a_ values. For the Cα of isoAsp (Eqs. 3,4), δ_obs_ is the pH-dependent chemical shift, and δ_min_ and δ_max_ correspond to the chemical shifts of the fully protonated and fully deprotonated form, respectively. As Asp_C-term_ (Eqs. 1,2) has two ionizable groups, the equation considers two pK_a_ values (pK_a__1_ and pK_a__2_). For the Cα of Asp_C-term_, δ_obs_ is the pH-dependent chemical shift, and δ_min0_ and δ_max2_ correspond to the chemical shift of the fully protonated (C-terminal and side chain carboxylic group) and fully deprotonated form (C-terminal and side chain carboxylic group), respectively. δ_max1_ and δ_min1_ are the chemical shifts of the fully protonated side chain and fully deprotonated C-terminal carboxylic group, respectively. The pK_a_ values were calculated using the software ORIGIN Pro 2019 (OriginLab Corporation) using the following functions:

1 $${\updelta }_{{obs}}={\updelta }_{min0}\frac{{\updelta }_{max1}-{\updelta }_{min0}}{1+{10}^{(pK_{a1}-pH)}}+\frac{{\updelta }_{max2}-{\updelta }_{min1}}{1+{10}^{(pK_{a2}-pH)}} ({\delta_{acid} }<{ \delta_{base}})$$

2 $${\updelta }_{{obs}}={\updelta }_{min0}\frac{{\updelta }_{max1}-{\updelta }_{min0}}{1+{10}^{(pH-pK_{a1})}}+\frac{{\updelta }_{max2}-{\updelta }_{min1}}{1+{10}^{(pH-pK_{a2})}} ({\delta_{acid}}>{ \delta_{base}})$$

3 $${\updelta }_{{obs}}={\updelta }_{max}+ \frac{{\updelta }_{min}-{\updelta }_{max}}{1+{10}^{(pH-{pK}_{a})}} ({\delta_{acid}}<{ \delta_{base}})$$

4 $${\updelta }_{{obs}}={\updelta }_{min}+ \frac{{\updelta }_{max}-{\updelta }_{min}}{1+{10}^{(pH-{pK}_{a})}} ({\delta_{acid}}>{ \delta_{base}})$$

## Supplementary Information

Below is the link to the Supplementary Information.

Supplementary Information 1 (PDF 3013 kb)
