# Supplementary material for: Detecting aspartate isomerization and backbone cleavage after aspartate in intact proteins by NMR spectroscopy
Source: J Biomol NMR. 2021 Jan 21;75(1):71–82. doi: 10.1007/s10858-020-00356-4 (PMC7897204; doi:10.1007/s10858-020-00356-4)
Supplement: Supplementary file 1 — Supplementary Information 1 (PDF 3013 kb) [file 10858_2020_356_MOESM1_ESM.pdf]

Supplementary Information

Detecting aspartate isomerization and backbone cleavage after aspartate in intact proteins by NMR spectroscopy

AUTHORS:

Arthur Hinterholzer, Vesna Stanojlovic, Christof Regl, Christian G. Huber, Chiara Cabrele and Mario Schubert

Supplementary Figures

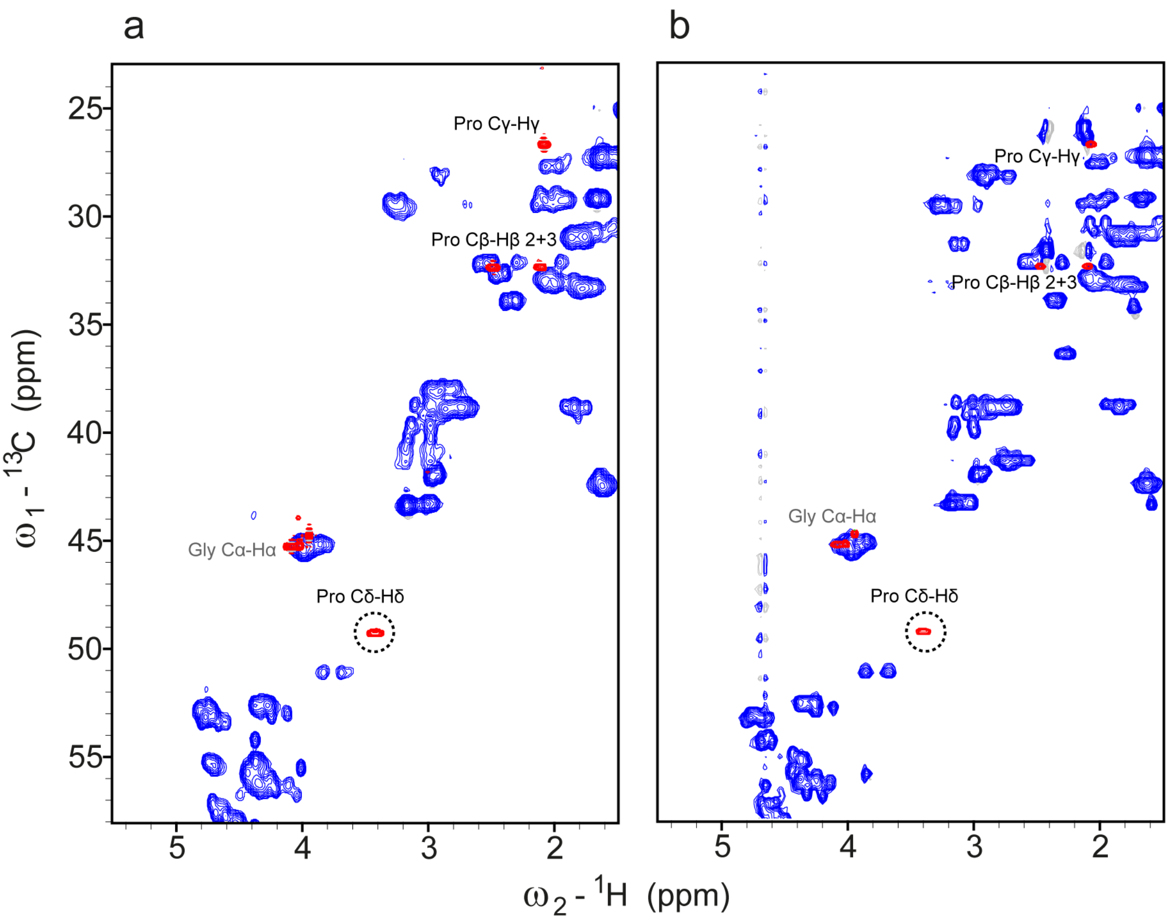

Supplementary Figure S1: Random coil chemical shifts of the natural amino acids compared with the reference random coil chemical shifts of $Pro_{N\text{-}term}$. (a) ^{1}H-^{13}C HSQC spectra of lysozyme and H-Pro-Gly-Gly-Gly-NH_2 at pH 2.3. (b) Comparable spectra at pH 7.4. The reference peptide H-Pro-Gly-Gly-Gly-NH_2 (red) shows at both pH values an unique cross peak that corresponds to Pro Cδ-Hδ compared with denatured lysozyme (blue).

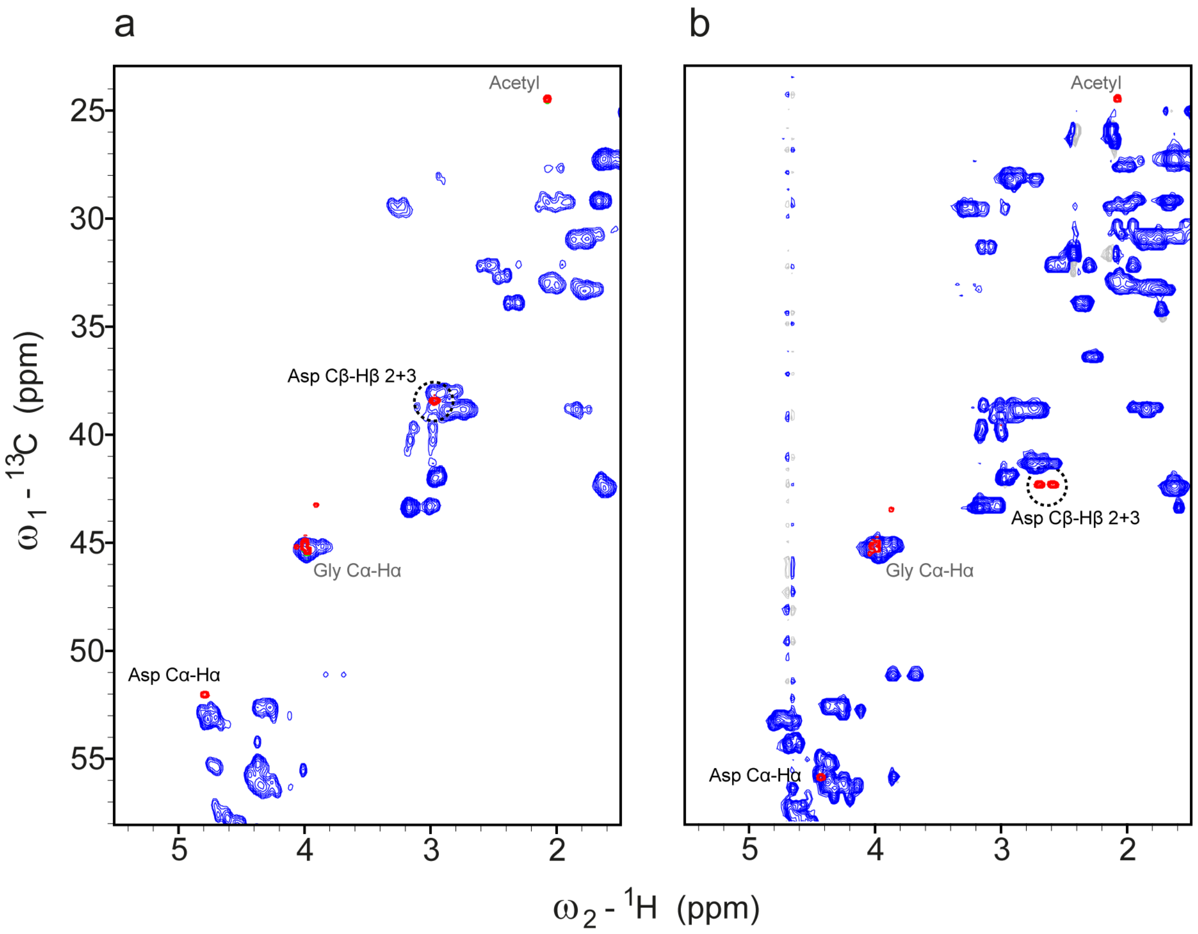

Supplementary Figure S2: Random coil chemical shifts of the natural amino acids compared with the reference random coil chemical shifts of $Asp_{C\text{-}term}$ at pH 2.3 and 7.4. a) Overlay of ^{1}H-^{13}C HSQC spectra of lysozyme (blue) under denaturing conditions (7 M urea-d_4 +D_2O) at pH 2.3 with the reference peptide Ac-Gly-Gly-Gly-Asp-OH (red). At this condition there are no Asp peaks completely isolated, only Asp Cα-Hα gives an indication for C-terminal Asp. b) Comparable pectra at a pH of 7.4. Here the C-terminal Asp Cβ-Hβ2+3 correlations shifted to an unique position and are therefore suitable for detection and quantification of Asp Pro cleavage.

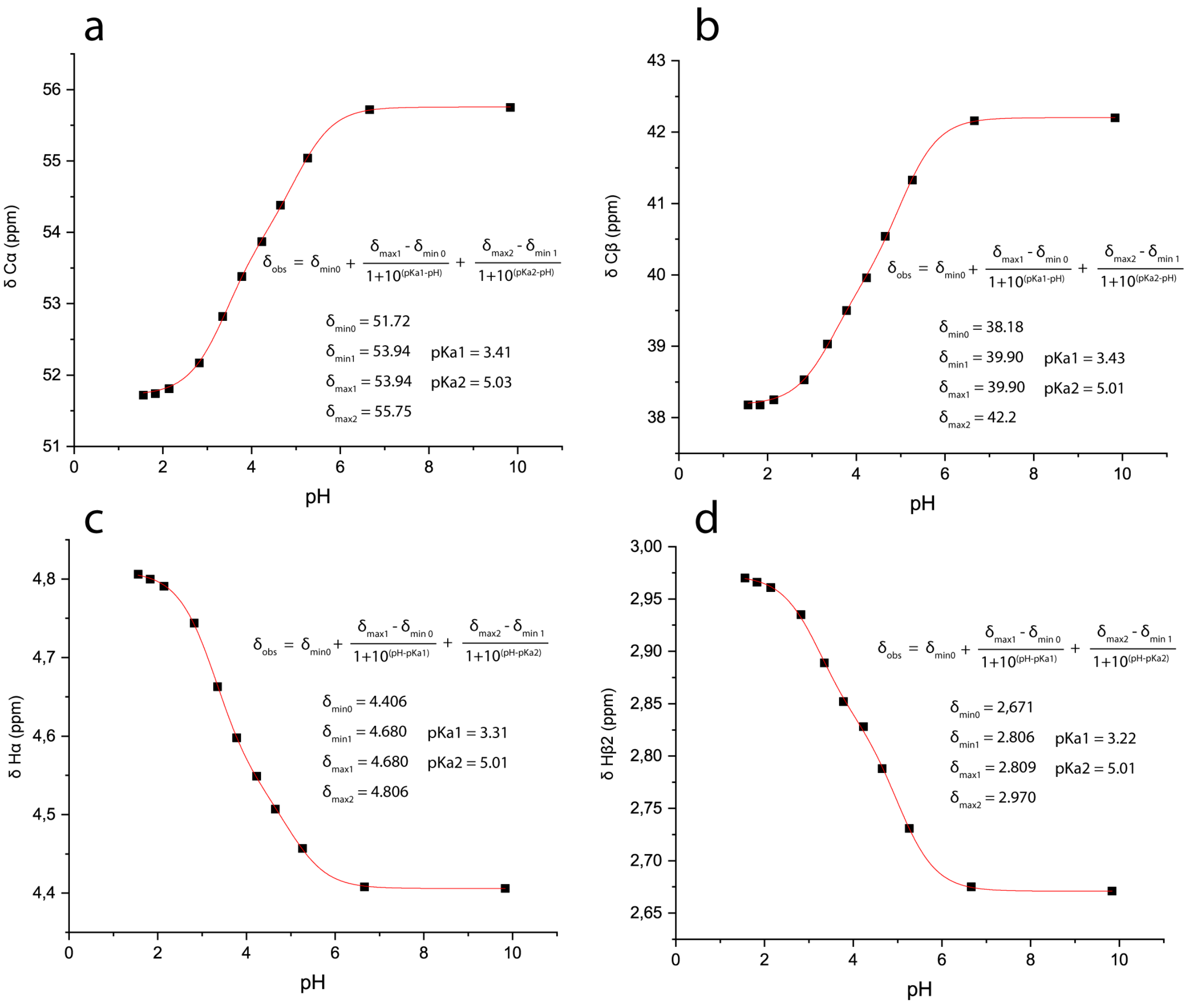

Supplementary Figure S3: Four titration curves using the chemical shifts (Hα, Cα, Hβ2, and Cβ) of $Asp_{C\text{-}term}$ (Table S2) at different pH values to determine its pK_a value using a small peptide (Ac-Gly-Gly-Gly-Asp-OH).

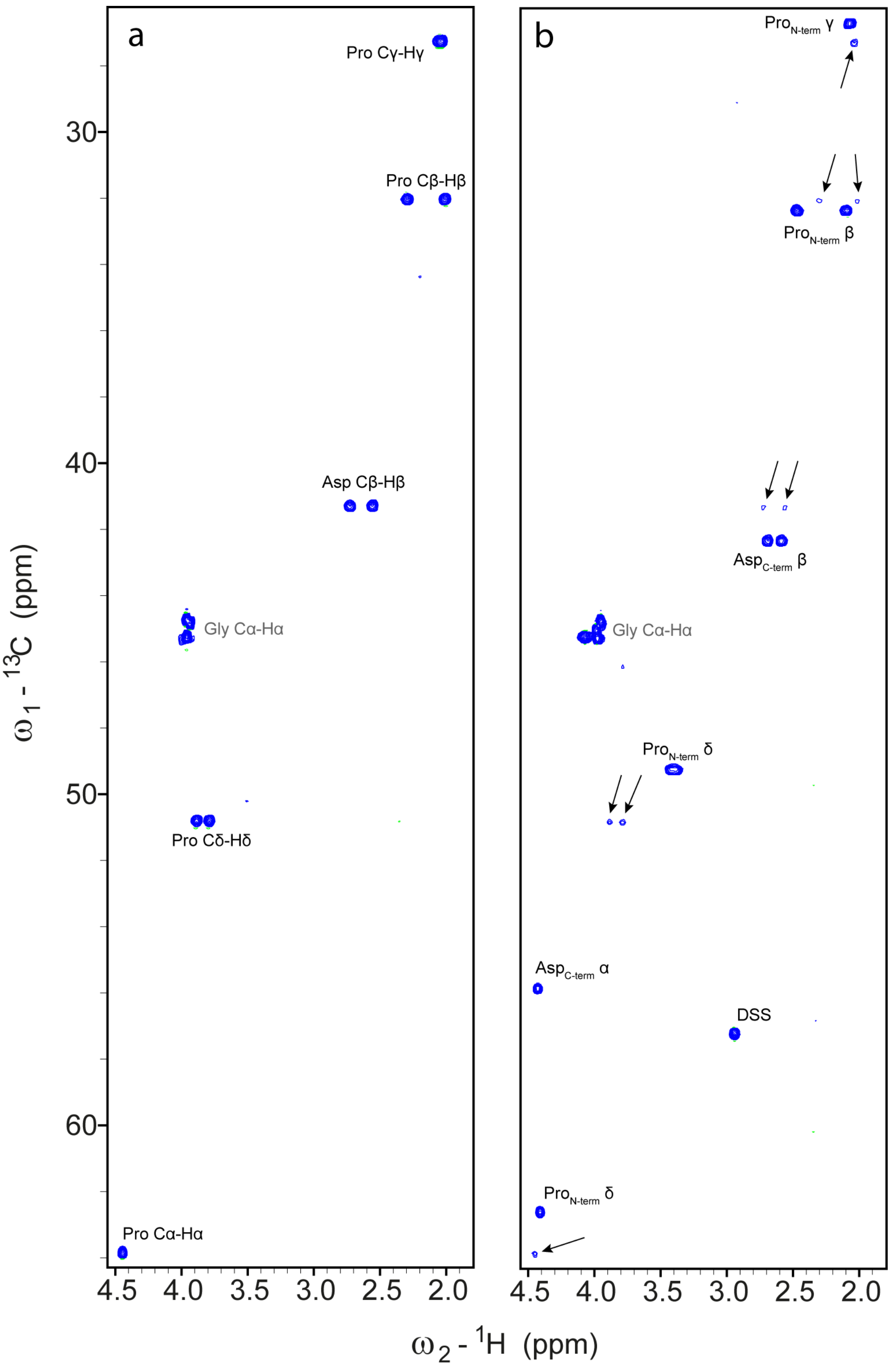

Supplementary Figure S4: ^1H-^{13}C HSQC spectra of the peptide Ac-Gly-Gly-Asp-Pro-Gly-Gly-NH_2 with and without treatment at pH 2.6 (25 h at 60°C). a) Spectrum of the untreated peptide (7 M urea-d_4 in D_2O, pH adjusted to 7.4, NS: 90, 512×256 complex points, relaxation delay: 1.3 s, runtime: 18 h). b) Spectrum of the treated peptide (7 M urea-d_4 + D_2O, pH adjusted to 7.4, NS: 70, 512×256 complex points, relaxation delay: 1.3 s, runtime: 14 h). The arrows point to the original non-terminal Asp/Pro signals.

Rituximab heavy chain

```
        10         20         30         40         50         60
QVQLQQPGAE LVKPGASVKM SCKASGYTFT SYNMHWVKQT PGRGLEWIGA IYPGNGDTSY
        70         80         90        100        110        120
NQKFKGKATL TADKSSSTAY MQLSSLTSED SAVYYCARST YYGGDWYFNV WGAGTTVTVS
       130        140        150        160        170        180
AASTKGPSVF PLAPSSKSTS GGTAALGCLV KDYFPEPVTV SWNSGALTSG VHTFPAVLQS
       190        200        210        220        230        240
SGLYSLSSVV TVPSSSLGTQ TYICNVNHKP SNTKVDKKAE PKSCDKTHTC PPCPAPELLG
       250        260        270        280        290        300
GPSVFLFPPK PKDTLMISRT PEVTCVVVDV SHEDPEVKFN WYVDGVEVHN AKTKPREEQY
       310        320        330        340        350        360
NSTYRVVSVL TVLHQDWLNG KEYKCKVSNK ALPAPIEKTI SKAKGQPREP QVYTLPPSRD
       370        380        390        400        410        420
ELTKNQVSLT CLVKGFYPSD IAVEWESNGQ PENNYKTTPP VLDSDGSFFL YSKLTVDKSR
       430        440        450
WQQGNVFSCS VMHEALHNHY TQKSLSLSPG (K)
```

Rituximab light chain

```
        10         20         30         40         50         60
QIVLSQSPAI LSASPGEKVT MTCRASSSVS YIHWFQQKPG SSPKPWIYAT SNLASGVPVR
        70         80         90        100        110        120
FSGSGSGTSY SLTISRVEAE DAATYYCQQW TSNPPTFGGG TKLEIKRTVA APSVFIFPPS
       130        140        150        160        170        180
DEQLKSGTAS VVCLLNNFYP REAKVQWKVD NALQSGNSQE SVTEQDSKDS TYSLSSTLTL
       190        200        210
SKADYEKHKV YACEVTHQGL SSPVTKSFNR GEC
```

Supplementary Figure S5: Sequence of the therapeutic mAb rituximab with the unique Asp-Pro sequence highlighted.

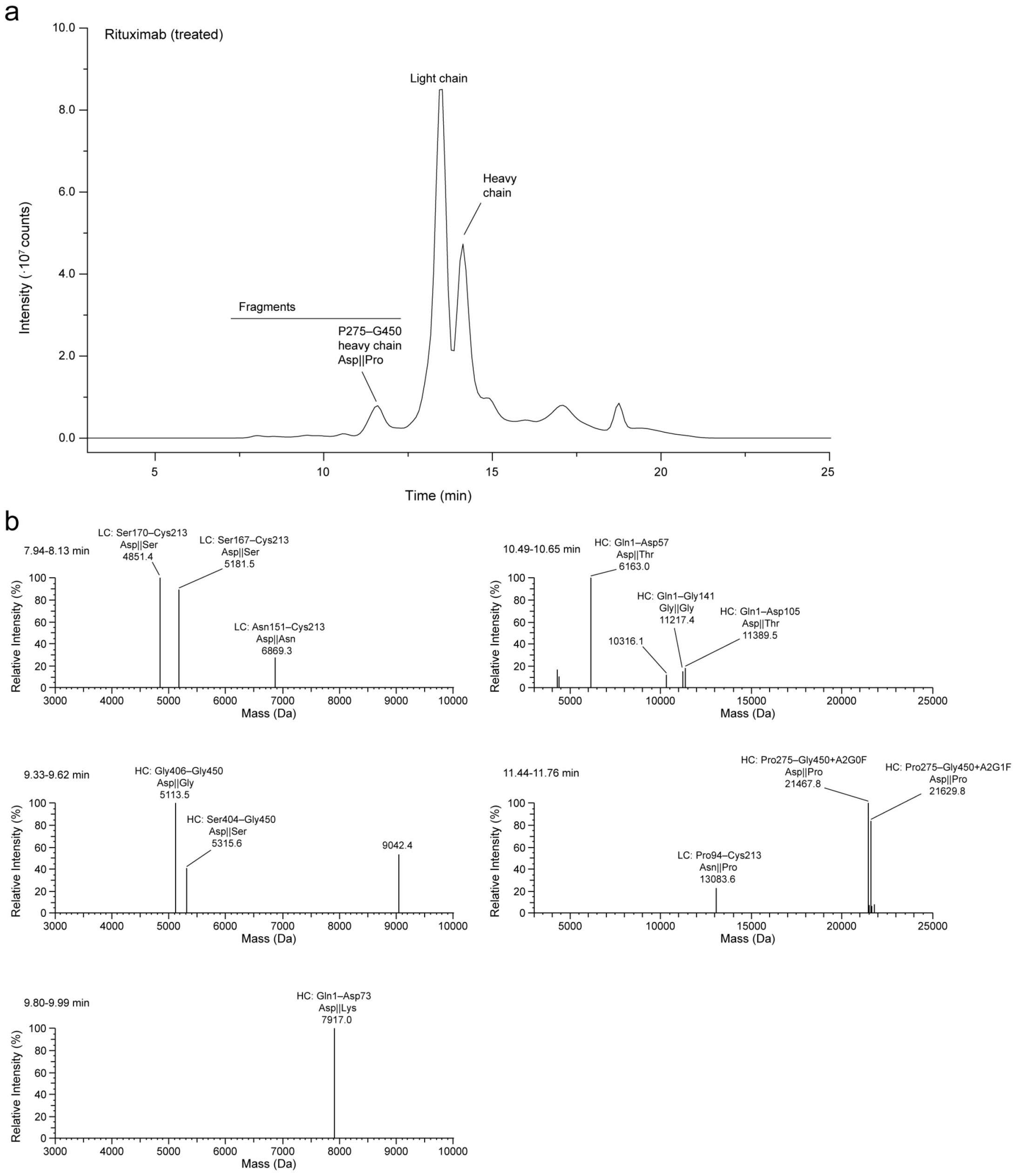

Supplementary Figure S6: HPLC-MS analysis of rituximab treated at pH 4 and after reducing the disulfides with 5mmol/L TCEP. a) Total ion current chromatogram (TICC) showing that the main cleavage occurs between Asp274 and Pro275. b) Deconvoluted annotated spectra at the indicated retention times showing strand cleavage in the mAb.

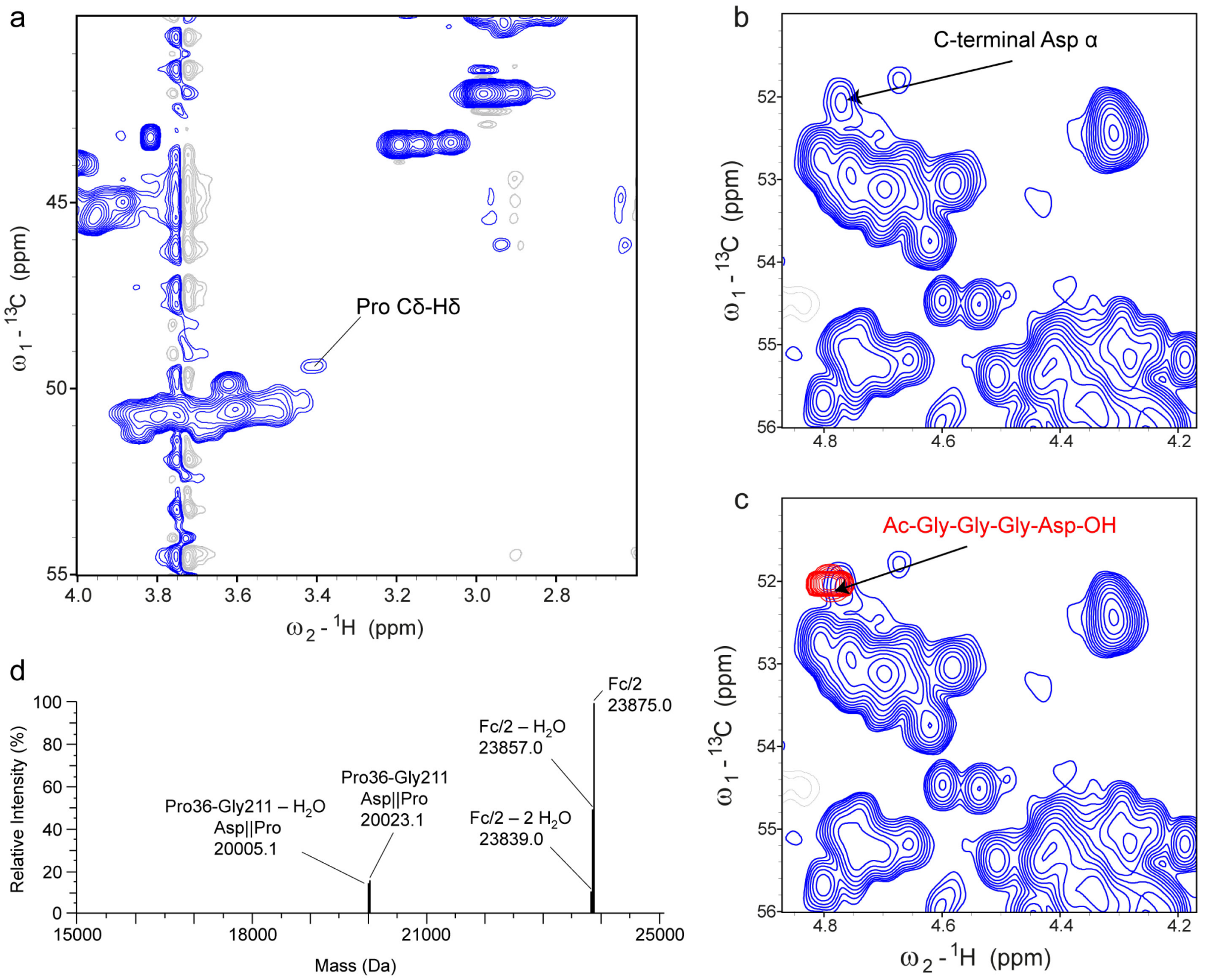

Supplementary Figure S7: Backbone cleavage between Asp and Pro in treated recombinant Fc/2 protein. a-c) Regions of a ^{1}H-^{13}C HSQC spectrum of Fc/2 showing Cδ-Hδ correlations of $Pro_{N\text{-term}}$ which was prepared and measured as described in Grassi et. al. (Grassi et al. 2017). d) Deconvoluted annotated spectrum of the reduced Fc/2 protein showing strand cleavage in Fc/2 (Pro36-Gly211 in Fc/2 corresponds to Pro275-Gly450 in the heavy chain of rituximab).

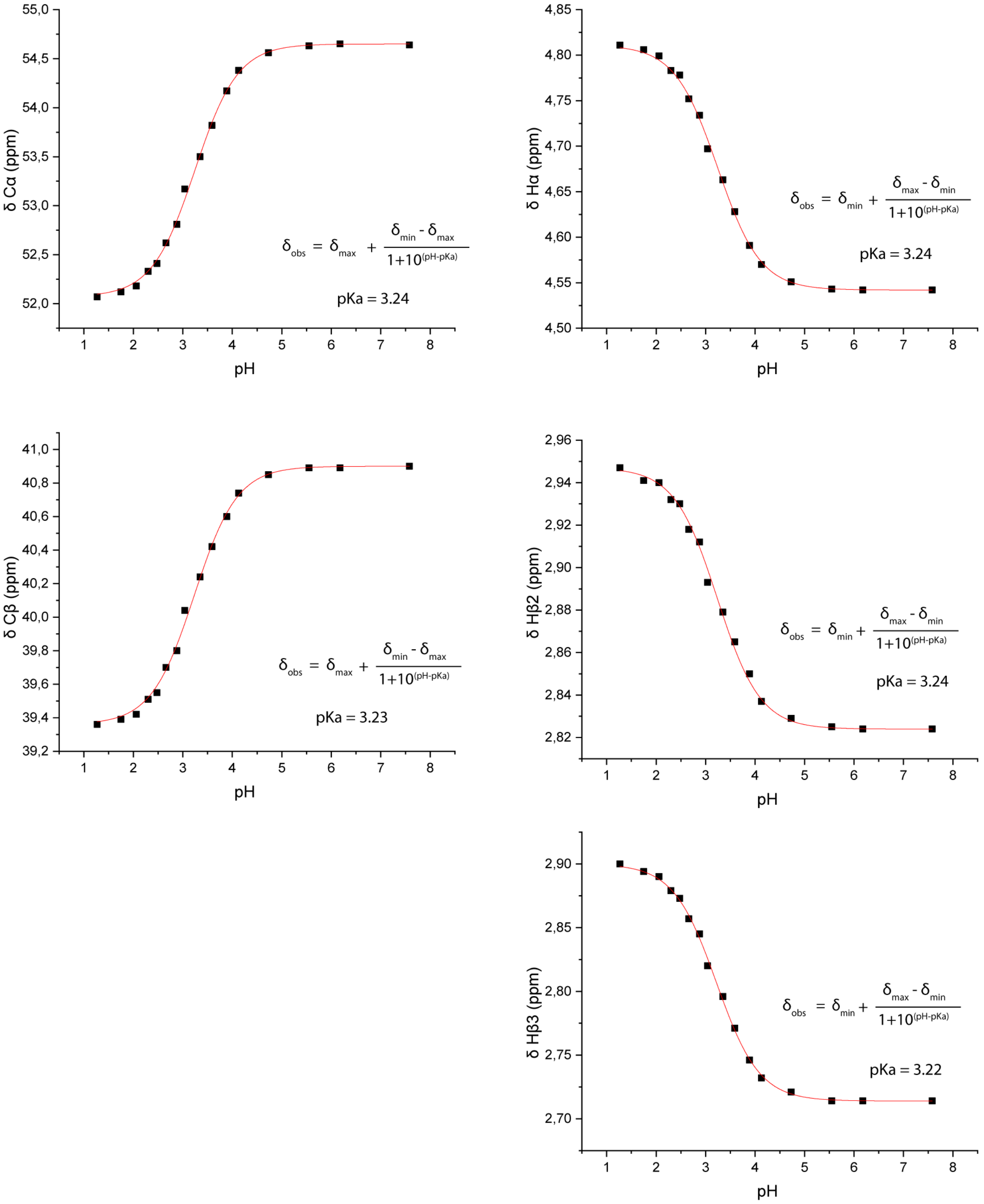

Supplementary Figure S8: Five titration curves using the chemical shifts (Hα, Cα, Hβ2, Hβ3 and Cβ) of isoAsp (Table S1) at different pH values to determine its pK_a value using a small peptide (Ac-Gly-Gly-isoAsp-Gly-Gly-NH_2)

Supplementary Tables:

Supplementary Table S1: Synthesized peptides used to determine the random-coil chemical shifts of $Pro_{N\text{-}term}$, $Asp_{C\text{-}term}$ and isoAsp.

Number	**Peptide sequence**	**M_{found} (M_{calcd}) / Da**[a]	**t_R/min**[b]
1	*Ac*-Gly-Gly-Gly-Asp-*OH*	346.46 (346.30)	4.51/4.89[c] (80%)
2	*H*-Pro-Gly-Gly-Gly-*NH2*	286.38 (285.30)	2.89 (92%)
3	*Ac*-Gly-Gly-Asp-Pro-Gly-Gly-*NH2*	499.61 (499.48)	13.59 (92%)
4	*Ac*-Gly-Gly-isoAsp-Gly-Gly-*NH2*	401.24 (402.36)	2.79 (80%)

[a] Positive ion mode MALDI-TOF-MS. Matrix: HCCA. [b] HPLC gradient: 1% B for 8 min, 1-50% B over 35 min. [c] Eluted as double peak.

Supplementary Table S2: Chemical shift values of C-terminal Asp dependent on the pH for the calculation of its pK_a value in a small peptide (Ac-Gly-Gly-Gly-Asp-OH). The peptide was dissolved in D_2O and the pH values were adjusted with DCl and NaOD.

pH	Cα	Hα	Cβ	Hβ2	Hβ3
1.56	51.72	4.806	38.18	2.970	2.970
1.83	51.74	4.800	38.18	2.966	2.966
2.14	51.81	4.791	38.25	2.961	2.961
2.82	52.17	4.744	38.53	2.935	2.935
3.35	52.82	4.663	39.03	2.889	2.889
3.78	53.38	4.598	39.50	2.852	2.834
4.23	53.87	4.549	39.96	2.828	2.779
4.65	54.38	4.507	40.54	2.788	2.716
5.26	55.04	4.457	41.33	2.731	2.631
6.66	55.72	4.408	42.16	2.675	2.544
9.83	55.75	4.406	42.2	2.671	2.538

Supplementary Table S3. Identified mAb fragments of rituximab treated at pH 4 and analyzed by mass spectrometry after reducing the disulfides with 5mmol/L TCEP

Retention time (min)[a]	Fragment[b]	Cleavage[c]	Mass (Da)[d]	Theoretical mass (Da)[e]	Δppm[f]	Intensity
7.94–8.13	LC: Ser170–Cys213	Asp||Ser	4851.4	4851.4	4.1	36632
7.94–8.13	LC: Ser167–Cys213	Asp||Ser	5181.5	5181.5	4.3	32643
7.94–8.13	LC: Asn151–Cys213	Asp||Asn	6869.3	6869.2	4.6	10182
9.33–9.62	HC: Gly406–Gly450	Asp||Gly	5113.5	5113.5	5.3	12283
9.33–9.62			9042.4			6608
9.33–9.62	HC: Ser404–Gly450	Asp||Ser	5315.6	5315.6	4.6	5079
9.80–9.99	HC: Gln1–Asp73	Asp||Lys	7917.0	7916.9	5.0	25852
10.49–10.65	HC: Gln1–Asp57	Asp||Thr	6163.0	6163.0	4.8	50090
10.49–10.65	HC: Gln1–Asp105	Asp||Trp	11389.5	11389.4	5.0	9086
10.49–10.65			4301.2			8409
10.49–10.65	HC: Gln1–Gly103	Gly||Gly	11217.4	11217.4	4.7	7667
10.49–10.65			10316.1			6034
10.49–10.65			4388.2			5359
11.44–11.76	HC: Pro275–Gly450 + A2G0F	Asp||Pro	21467.8	21467.6	7.2	**80543**
11.44–11.76	HC: Pro275–Gly450 + A2G1F	Asp||Pro	21629.8	21629.7	6.3	**67712**
11.44–11.76	LC: Pro94–Cys213	Asn||Pro	13083.6	13083.5	4.7	18362
11.44–11.76	HC: Pro275–Gly450 + A2G0F - H_2O	Asp||Pro	21449.7	21449.6	6.1	14459
11.44–11.76	HC: Pro275–Gly450 + A2G1F - H_2O	Asp||Pro	21611.8	21611.6	6.0	12463
11.44–11.76			21483.7			7947
11.44–11.76			21790.9			6746
11.44–11.76			21499.7			5855
11.44–11.76			21645.8			5553

[a]Retention time used for averaging spectra for deconvolution of mass spectra

[b]Identified fragments (HC= heavy chain, LC= light chain)

[c]Cleavage site (|| marks the cleavage of the peptide bond)

[d]Experimentally obtained monoisotopic mass

[e]Theoretical mass determined by GPMAW (Version 9.51) (citation: Trends in Biochemical Sciences, 01 Nov 2001, 26(11):687-689; DOI: 10.1016/s0968-0004(01)01954-5)

[f]Deviation of experimentally determined and theoretical mass in ppm

Supplementary Table S4. Identified fragments in recombinantly produced Fc/2 treated at pH 4 and analyzed by mass spectrometry

Retention time (min)[a]	Fragment[b]	Cleavage[c]	Mass (Da)[d]	Theoretical mass (Da)[e]	Δppm[f]	Intensity[g]
7.56–8.11	Fc/2		23875.0	23875.0	1.7	26300
7.56–8.11	Fc/2- H_2O		23857.0	23857.0	1.4	13000
7.56–8.11	Pro36-Gly211	Asp||Pro	20023.1	20023.1	3.7	4130
7.56–8.11	Pro36-Gly211- H_2O	Asp||Pro	20005.1	20005.1	0.0	3860
7.56–8.11	Fc/2+ O		23891.0	23891	0.4	3250
7.56–8.11	Fc/2 - 2x H_2O		23839.0	23839	1.0	2700

[a]Retention time used for averaging spectra for deconvolution of mass spectra

[b]Identified fragments

[c]Cleavage site (|| marks the cleavage of the peptide bond)

[d]Experimentally obtained monoisotopic mass

[e]Theoretical mass determined by GPMAW (Version 9.51)

[f]Deviation of experimentally determined and theoretical mass in ppm

Supplementary Table S5: Chemical shift values of isoAsp dependent on the pH for the calculation of its pK_a value in a small peptide (Ac-Gly-Gly-isoAsp-Gly-Gly-NH_2). The peptide was dissolved in D_2O and the pH values were adjusted with DCl and NaOD.

pH	Cα	Hα	Cβ	Hβ2	Hβ3
1.27	52.07	4.811	39.36	2.947	2.900
1.75	52.12	4.806	39.39	2.941	2.894
2.06	52.18	4.799	39.42	2.940	2.890
2.30	52.33	4.783	39.51	2.932	2.879
2.48	52.41	4.778	39.55	2.930	2.873
2.66	52.62	4.752	39.70	2.918	2.857
2.88	52.81	4.734	39.80	2.912	2.845
3.04	53.17	4.697	40.04	2.893	2.820
3.35	53.50	4.663	40.24	2.879	2.796
3,59	53.82	4.628	40.42	2.865	2.771
3.89	54.17	4.591	40.60	2.850	2.746
4.13	54.38	4.570	40.74	2.837	2.732
4.73	54.56	4.551	40.85	2.829	2.721
5.55	54.63	4.543	40.89	2.825	2.714
6.18	54.65	4.542	40.89	2.824	2.714
7.58	54.64	4.542	40.90	2.824	2.714

Supplementary Table S6: Chemical shift values of $Asp_{c\text{-}term}$ observed in a therapeutic mAb at pH 7.4 in comparison to values obtained from Ac-Gly-Gly-Gly-Asp-OH.

Resonance	Ac-Gly-Gly -Gly-Asp-OH	Rituximab (treated)
Cα	55.9	n.d.
Hα	4.43	n.d.
Cβ	42.3	42.4
Hβ2	2.69	2.70
Hβ3	2.59	2.60
